# Early deformation mechanisms in the shear affected region underneath a copper sliding contact

**DOI:** 10.1038/s41467-020-14640-2

**Published:** 2020-02-11

**Authors:** C. Haug, F. Ruebeling, A. Kashiwar, P. Gumbsch, C. Kübel, C. Greiner

**Affiliations:** 1 Karlsruhe Institute of Technology (KIT), Institute for Applied Materials (IAM), Kaiserstrasse 12, 76131 Karlsruhe, Germany; 2 KIT IAM-CMS MicroTribology Center (µTC), Strasse am Forum 5, 76131 Karlsruhe, Germany; 3 Karlsruhe Institute of Technology (KIT), Institute of Nanotechnology, Hermann-von-Helmholtz-Platz 1, 76344 Eggenstein-Leopoldshafen, Germany; 4 Department of Materials and Earth Sciences, Technical University of Darmstadt (TUD), 64287 Darmstadt, Germany; 5 Fraunhofer Institute for Mechanics of Materials (IWM), Woehlerstrasse 11, 79108 Freiburg, Germany; 6 Karlsruhe Nano Micro Facility, Karlsruhe Institute of Technology (KIT), 76344 Eggenstein-Leopoldshafen, Germany

**Keywords:** Structural materials, Surfaces, interfaces and thin films

## Abstract

Dislocation mediated plastic deformation decisively influences the friction coefficient and the microstructural changes at many metal sliding interfaces during tribological loading. This work explores the initiation of a tribologically induced microstructure in the vicinity of a copper twin boundary. Two distinct horizontal dislocation traces lines (DTL) are observed in their interaction with the twin boundary beneath the sliding interface. DTL formation seems unaffected by the presence of the twin boundary but the twin boundary acts as an indicator of the occurring deformation mechanisms. Three concurrent elementary processes can be identified: simple shear of the subsurface area in sliding direction, localized shear at the primary DTL and crystal rotation in the layers above and between the DTLs around axes parallel to the transverse direction. Crystal orientation analysis demonstrates a strong compatibility of these proposed processes. Quantitatively separating these different deformation mechanisms is crucial for future predictive modeling of tribological contacts.

## Abstract (web summary)

The microstructural evolution during the sliding of two surfaces against each other is complex and remains poorly understood. Here, the authors use electron microscopy to elucidate the different deformation mechanisms occurring at the beginning of sliding.

## Introduction

Tribology studies friction, wear, and lubrication of surfaces interacting in relative motion^1^. Recent studies affirm the substantial impact friction and wear have on global energy consumption, e.g. in the transportation sector^2^. This corroborates the importance of basic and applied research in the field of materials tribology. Tribological loading commonly causes plastic deformation and microstructural changes in the subsurface area of one or both bodies constituting a sliding interface^3,4^. The modified material’s composition can be structurally and chemically different from that of either bulk material^5,6^. These microstructural modifications alter the properties of the material in the subsurface area and in turn affect friction and wear^7–9^. However, the underlying elementary deformation mechanisms are not sufficiently clear to allow for systematic modifications or even predictive simulation^10^. Atomistic simulations in nanocrystalline structures have demonstrated that the inhomogeneities of plastic deformation in the different grains and grain rotation play a significant role in tribologically induced deformation and wear^11^. Experimentally, the elementary mechanisms governing the early stages of tribological loading have been studied less extensively, even though they seem elemental in deciding the future fate of a tribological contact. We have previously reported a distinct horizontal line feature at a uniform depth of about 150 nm below the surface of copper samples contacted in dry sliding conditions by a sapphire sphere^12^. Since this line was found to consist of dislocations, it was named dislocation trace line (DTL). The DTL is the first discontinuity within the microstructure and therefore decisive for further microstructure evolution^4,12^.

In the present study, we investigate the interaction of the DTL with a coherent ∑3 twin boundary (TB) after a single sliding pass on high-purity copper. Detailed (scanning) transmission electron microscopy investigations including automated crystal orientation mapping (ACOM) shed light on the characteristics of this horizontal microstructural discontinuity. They allow to decipher the characteristics of the plastic deformation occurring during early-stage sliding beneath the sliding interface. Three distinct concurrent shearing and grain rotation processes are identified.

## Results

### Microstructure

**Fig. 1:**
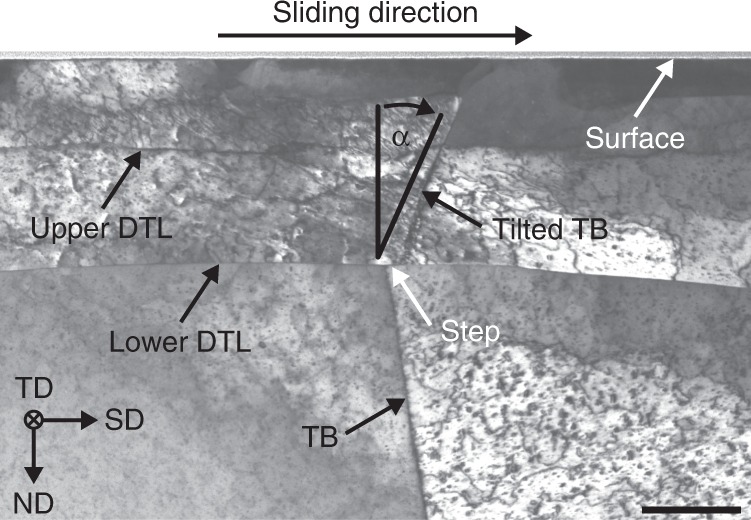
STEM bright-field (BF) image of tribologically deformed microstructure after a single sliding pass in the vicinity of a twin boundary (TB). Directly above the surface, the first protective platinum layer is visible. The TB as well as the upper and lower dislocation trace lines (DTL) are marked by arrows. Both DTLs extend through the TB to either of its sides. At the intersection of the TB with the lower DTL, a displacement step in sliding direction (SD) is visible. The angle *α* denotes the inclination between the sample surface normal direction and the TB segments separated by the DTLs. Sliding direction (SD), normal (force) direction (ND) and transverse direction (TD) form the right-handed sample coordinate system indicated in the image and used throughout this work. The scale bar corresponds to 200 nm.

The copper subsurface microstructure after a single sliding pass is shown in the STEM image in Fig. 1. Two DTLs are observed, visible as sharp discontinuities parallel to the sliding interface, exactly as previously described for much lower normal loads^4,12^. Both trace lines traverse a pre-existing TB at constant depths of approximately 185 and 415 nm from the sample surface. From now on they will be referred to as upper and lower DTL. The microstructure exhibits a decreasing density of defects visible in STEM BF contrast with increasing depth (see also Supplementary Fig. 1 with a much lower magnification). The average friction coefficient measured during the sliding pass was *µ* = 0.34.

The segment of the TB in the bulk, i.e. below the lower DTL, is inclined at an angle of *α* ≈ −9° with respect to the normal of the sample surface in the cross-sectional view, as denoted in Fig. 1. This inclination angle abruptly changes to a value of *α* ≈ 20° at the intersection of the TB with the lower DTL, thereby preserving the straight line shape of the boundary present in the bulk, which exhibits only negligible curvature. The TB segment above the lower DTL can be described as being tilted into the direction of sliding (SD) by an angle of 29° with respect to the bulk part. The segment above the upper DTL is tilted even further by an additional angle of 2° (*α* ≈ 22°). This abrupt change of the boundary’s alignment coincides with a displacement step of the TB along the lower DTL in SD. The length of this displacement measures ~40 nm. The area above the upper DTL in Fig. 1 entails distinct areas enclosed by sharp contrast changes directly adjacent to the sliding interface. To further characterize these areas, the crystallographic orientation is measured by ACOM.

### Crystallographic orientations

**Fig. 2:**
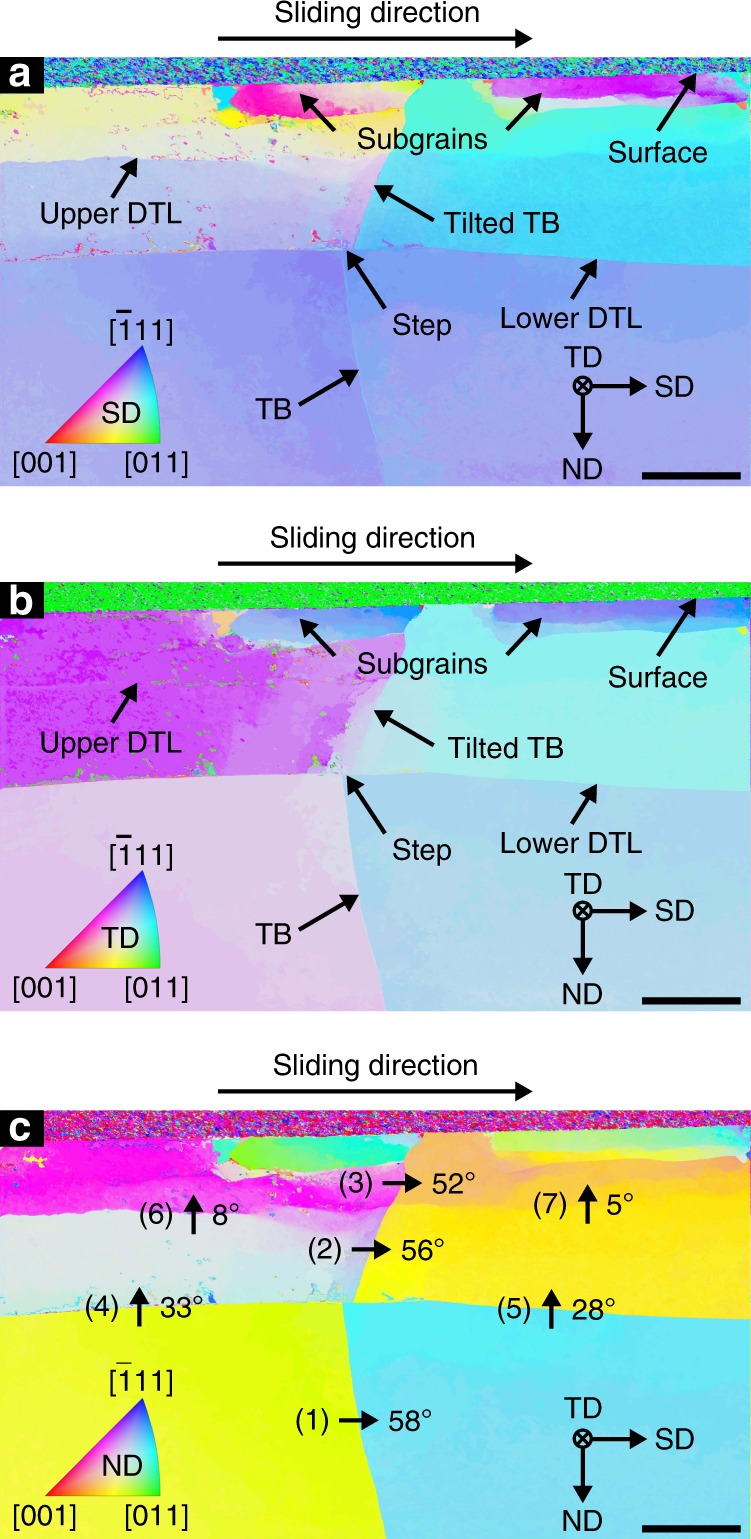
Crystallographic orientation data (ACOM) of tribologically deformed microstructure after a single sliding pass in the vicinity of the TB. **a** Crystallographic orientation data plotted using the depicted inverse pole figure (IPF) color key with respect to SD. Orientation changes at both DTLs as well as orientation gradients within the subsurface subgrains are visible. **b** Crystallographic orientation data plotted with TD color coding. The absence of a distinct orientation (i.e. color) change at the upper DTL is of special note. **c** Crystallographic orientation data plotted with ND color coding. Arrows 1–7 denote the magnitude of the misorientation angle between the two points at the start and end of each arrow. Each given misorientation angle corresponds to the specific misorientation (of all crystallographically equivalent misorientations) possessing the smallest misorientation angle for the two orientations considered. A misorientation decrease at the TB from bulk to subsurface area (arrows 1–3) is apparent. The lower DTL (arrows 4 and 5) accommodates a smaller misorientation than the upper DTL (arrows 6 and 7). At both DTLs, the misorientation is smaller at the segment right of the TB (arrows 5 and 7) as compared to the segment left of the TB (arrows 4 and 6). The surface normal orientations (i.e. inverse of ND) left and right of the TB in the original state below the lower DTL (start and end of arrow 1) are approximately (−4, 7, 0) and (5, −4, −2) in crystal coordinates (Miller indices). After loading this changed to approximately (−12, 4, 5) and (4, −12, 1), measured above the upper DTL (start and end of arrow 3). All scale bars correspond to 250 nm.

Figure 2a displays the spatially resolved crystallographic orientations of the copper lattice in the vicinity of the TB colored with respect to SD (cf. raw data^13^). A standard inverse pole figure color key for the face-centered cubic (fcc) fundamental sector (Fm-3m symmetry) as depicted in Fig. 2a is used throughout the paper, associating crystallographic orientations with colors. The distinct subsurface areas visible in Fig. 1 are identified as subgrains, characterized by sharp transitions of the crystallographic orientation around their circumscription. An orientation gradient with respect to SD is visible within the subgrains and—much less pronounced—left and right of the TB in the area between the two DTLs. Furthermore, both upper and lower DTL are accompanied by a sharp transition in crystal orientation with respect to SD. This transition is more pronounced for the lower DTL. Figure 2b depicts the same orientation data colored with regard to the transverse direction (TD). The orientation change at both DTLs is less distinct in TD, especially for the upper DTL, where it almost vanishes. This is not the case for the subgrain boundaries.

To allow a quantitative analysis of the crystal lattice misorientations at both DTLs and at the TB, numerical values for the angular misorientations between seven pairs of points in the microstructure (start and end of arrows 1–7) are provided in Fig. 2c. The orientation is color coded with respect to the direction in which the normal load was applied during the experiment (ND). Selected surface plane normal orientations are provided in the description of Fig. 2c. A graphical representation of misorientation variations along DTLs and TB is provided in Supplementary Fig. 2. This demonstrates that the point-by-point misorientation values given in Fig. 2c are each representative for their corresponding feature.

The observation of sharp transitions in the crystallographic orientation as well as orientation gradients within subgrains as previously stated for SD (Fig. 2a) are also visible in ND (Fig. 2c). Two additional observations are evident when considering the misorientations at the DTLs (Fig. 2c). First, the upper DTL accommodates a much smaller misorientation (8° and 5° at arrows 6 and 7) than the lower DTL (33° and 28° at arrows 4 and 5). Second, the misorientation at both DTLs is noticeably smaller on the right-hand side of the TB (arrows 5 and 7) than on the left-hand side of the TB (arrows 4 and 6). Arrows 1–3 in Fig. 2c denote the misorientations at the TB at different depths below the sliding interface. In the bulk (arrow 1), the TB misorientation angle of 58° differs only 2° from that of an ideal fcc twinning misorientation (60° rotation about 〈111〉/∑3). Above the lower DTL, the TB misorientation is decreased to 56° (arrow 2) and even further (to 52°) above the upper DTL (arrow 3). The TB segment above the lower DTL appears less sharp in the HRTEM images presented in Supplementary Fig. 3 in comparison to the TB segment in the bulk.

## Discussion

**Fig. 3:**
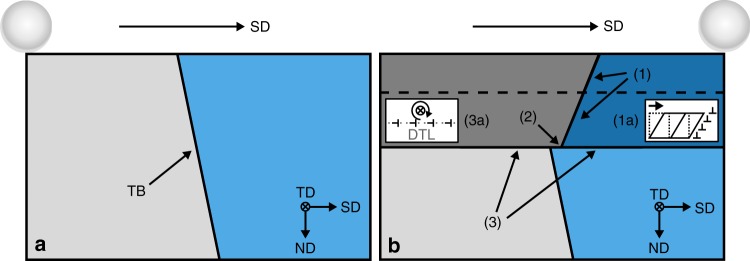
Model concept of key features and associated elementary processes occurring in the vicinity of the twin boundary (TB) during tribological loading. **a** Initial microstructure prior to tribological loading. In this state, the TB is assumed to possess a straight line shape all the way up to the sample surface (cf. Supplementary Fig. 4). **b** Simplified schematic of altered microstructure after passing of sphere including key features and governing processes. Microstructural changes at upper DTL (dashed line) are omitted for clarity. (1) The tilting of the TB segment observed above the lower DTL is attributed to a simple shear of the subsurface area, confined by the lower DTL. Inset (1a) visualizes the simple shear process and the character of edge dislocations involved. (2) A localized plastic shear process at the lower DTL is visible as step in sliding direction (SD). The TB may be regarded as a marker revealing both shear processes. (3) The lower DTL (horizontal line) accommodates a misorientation due to crystal lattice rotation of the whole volume above the DTL, depicted as a change in color. Inset (3a) visualizes the crystal rotation around an axis close to TD above the DTL and the character of exemplary edge dislocations accommodating the misorientation at the DTL.

For further analysis, the present observations regarding microstructure (Fig. 1) and crystal orientation (Fig. 2) after tribological loading are classified into three distinct features as depicted in the simplified schematic presented in Fig. 3. In the following paragraphs, each feature is successively discussed in detail. Elementary processes associated with and governing each feature’s characteristics are developed.

Figure 3a schematically depicts the microstructure before the experiment. Due to careful sample preparation, the initial defect density in the sample can be considered low and evenly distributed. This is reflected in the grains being homogenously colored. It is reasonable to assert that the TB under investigation is part of an annealing twin, which are commonly observed in annealed copper and possess a characteristic straight line shape^14,15^. In its initial state, it likely extended all the way up to the sample surface in an unperturbed straight line with an inclination *α* ≈ −9° against the surface normal as observed in the bulk area after the experiment (cf. Fig. 1). This assertion is additionally supported by the two STEM images presented in Supplementary Fig. 4, in which a tribologically sheared copper TB is compared to its undeformed state.

Figure 3b depicts a simplified model of the microstructure as observed after the present experiment (cf. Fig. 1) and illustrates the subsumption of observations under three key features: (1) a tilt of the TB in SD above the lower DTL, (2) a displacement step of the TB at the lower DTL, and (3) a sharp transition in crystal orientation at the (lower) DTL (cf. Fig. 2). The upper DTL (dashed line) is omitted in Fig. 3b for clarity. This seems reasonable since most observations and insights regarding feature (3) are also applicable to the upper DTL. Similarities and differences are explored after the discussion of the three features and their corresponding elementary processes.

In agreement with previous works^12^, some dislocation activity is also observed in the bulk (lower part of Fig. 1). However, plastic deformation is most pronounced directly above the lower DTL and decreases with depth from the surface. Correspondingly, orientation variations in the bulk are insignificant in comparison to the subsurface area (cf. Fig. 2), on which the present analysis consequently focuses. Some of the homogenously distributed, characteristic contrast variations in Fig. 1 may furthermore be attributed to FIB milling damage during TEM foil preparation^16^. Also, despite the propensity of copper to oxidize, evidence of tribologically induced oxidation processes are not observed in Fig. 1. This is in agreement with previous observations, where noticeable oxidation processes were first detected after 10 sliding cycles for a similar copper–sapphire system^17^. Static oxidation processes are considered irrelevant on the present time scale (cf. Fig. 5 in ref. ^18^). Therefore, the following analysis focuses on plastic deformation processes.

The tilting observed in Fig. 1 and labeled as feature (1) in Fig. 3b may be interpreted as the result of a simple shear process affecting the area confined between the sliding interface and the lower DTL. By applying a shear deformation to a volume containing a straight, vertical line feature like the TB, this feature would be tilted as observed in Fig. 1. An exemplary simple shear process is depicted in inset (1a) of Fig. 3b. While the dotted lines represent the undeformed volume, the solid lines show the volume after a simple shear deformation has been applied in the direction of the arrow. For the present experiment, dislocation movement within the copper crystal lattice is a plausible mode of plastic deformation^19,20^. More specifically, dislocations with an edge component of the effective burgers vector parallel to the sliding interface and moving in sliding direction in the volume between interface and the lower DTL (cf. Fig. 1) would be able to accommodate such a simple shear deformation. Exemplary suitable edge dislocations are depicted in inset (1a) of Fig. 3b.

Hamilton’s elastic theory of the stress field generated by a sliding sphere yields the stresses in the substrate^21^. The experimentally measured coefficient of friction (*µ* = 0.34) serves as an input parameter for the calculation of the traction component. This value is within the range of values of ~0.25–0.7 previously measured for comparable copper–sapphire systems, depending on the specific experimental conditions^4,12,22^. The resulting shear component of the stress tensor in SD provides a driving force enabling dislocation motion in the soft, initially not strain-hardened copper to accommodate the simple shear. Similar dislocation transport mechanisms have been previously reported in discrete dislocation simulations of a sliding spherical indenter^23^. The further increased inclination of the TB above the upper DTL (cf. Fig. 1) indicates that directly beneath the sliding interface, dislocation movement must have been even more pronounced, leading to an increased degree of shear. This may be rationalized considering that the shear stresses according to Hamilton’s theory are maximum at the surface for the given coefficient of friction^21^. It is important to note that without the presence of the TB, this shearing mechanism would likely have remained undetected. This is because the suspected dislocation movement accommodating the shear is not accompanied by the introduction of a crystal lattice misorientation but only a relative displacement of the crystal planes in SD. The dislocations responsible are not stationary and thus not present in the volume under consideration anymore during ex situ analysis, i.e. after the shear process has ceased. This renders them inaccessible to standard microscopic microstructure analysis. Thus, the TB may be considered a marker for the effects left behind by this simple shear process.

Grain boundaries may constitute obstacles for dislocation movement under certain conditions and influence the way dislocation slip propagates from one grain to another^24^. One may wonder about possible interactions between the dislocations accommodating the simple shear mechanism and the TB. Various criteria have been suggested in literature that allow a prediction of whether a certain dislocation is stopped at, transmitted through or incorporated into a certain grain boundary^25^. In order to apply any of those criteria, precise knowledge of the locally resolved (shear) stress state, dislocation character and grain boundary crystallography as well as geometry is required. The present experiment allows a rough estimate of the stress state using Hamilton’s theory^21^. This might however not be enough for a reliable quantitative analysis of individual slip systems and an unambiguous clarification of mechanisms such as (partial) absorption and emission or transmission of dislocations. Especially in the case of twin boundaries, the degree of resistance against slip transmission cannot easily be generalized, but may be high or low, depending on the type of dislocation^25–28^. Despite these complications, the microstructure near the TB as observed in Fig. 1 and Supplementary Fig. 3 suggests that the vast majority of dislocations contributing to the simple shear process effectively transmitted (at least partially) through the TB in SD, while a smaller number of dislocations may have been partially incorporated into the TB. The latter is visible as structural changes of the part of the TB above the lower DTL in Supplementary Fig. 3b, having experienced the most significant amount of shear. This is in agreement with a recent experimental investigation on slip transmission in copper bi-crystals by Malyar and co-workers, where slip transmission with only limited pile-up was dominant for multiple loading directions^26^.

Utilizing the TB as a marker enables a rough estimate of the magnitude of shear that occurred due to the tribological loading. Since the TB has retained a straight line shape after being sheared (cf. Figs. 1 and 2), it is reasonable to assume that dislocation paths accommodating the process were homogenously distributed vertically, i.e. across the height of the sheared area. The misorientation angle of about 30° between the TB segments below and above the lower DTL (cf. Fig. 1) implies that under assumption of a regular dislocation spacing, one edge dislocation (each displacing the lattice, the length of one burgers vector length in SD) is necessary on approximately every second lattice plane from the sliding interface to the lower DTL to accommodate the shear (1/tan(30°) ≈ 1.73). This interpretation is in agreement with an analysis of relevant distances measurements in the microstructure (cf. Supplementary Fig. 5).

In order to produce this high degree of shear, dislocation nucleation or multiplication is required. Where and how these processes occur can only be speculated, but roughness-assisted surface nucleation of dislocations (cf. ref. ^22^) is likely not solely responsible here.

The displacement step in SD at the lower DTL visible in Fig. 1 and labeled as feature (2) in Fig. 3b may be interpreted as a special case of feature (1). The step is attributed to a localized plastic shear process confined to the lower DTL with effectively the same type of dislocations. Motion of these dislocations in SD on the lower DTL displaces the whole subsurface area collectively with respect to the bulk. The displacement of grain boundaries induced by (shear) stresses or strain has been previously reported in literature (stress-/strain-induced grain boundary migration)^29,30^. However, since these grain boundary displacement mechanisms are usually observed in microcrystalline and nanocrystalline materials at elevated temperatures and appear in conjunction with grain coarsening, they are likely not decisive in the present case. Similar to process (1), this localized displacement step is only visible in the microstructure in Fig. 1 because of the TB’s presence. The displacement of 40 nm in SD theoretically corresponds to about 150 burgers vectors in SD for {111}〈110〉edge dislocations, assuming a copper lattice constant of 3.61 Å. The localization of this high a number of dislocations at the lower DTL may hint at a possible connection of the shear processes with the DTL’s formation and presence. For instance, it seems plausible that the DTL, once formed, acts as an obstacle to the shearing dislocations’ movement, confining the shear processes to the subsurface area above the lower DTL. More specifically, the DTL may accommodate dislocation movement in SD, similarly as has been reported for the movement of screw dislocations on planes located in parallel very closely to twin boundaries^25,31^, but prevent such movement in ND. Dislocations impinging onto the DTL under an arbitrary angle might dissociate into a glissile part moving in SD, contributing to the localized shear, and an immobile part, incorporated into the DTL.

In order to further investigate this notion, feature (3) in Fig. 3b needs to be considered in detail: The sharp transition in crystal orientation at both DTLs (cf. Fig. 2a–c) indicates that the DTLs accommodate the crystal misorientations between the areas they separate in a similar way grain boundaries do, i.e. in a very narrow region in their immediate vicinity. In conjunction with the relatively uniform crystal orientation within all six areas of the microstructure separated by the two DTLs and the TB (Fig. 2a–c), it seems reasonable to assume that the entire region above the lower DTL has rotated as a whole with respect to the bulk, and the entire region above the upper DTL has rotated as a whole with respect to the region between the two DTLs (depicted as sharp color changes at the DTL in the model figure Fig. 3b). As indicated in Fig. 3a and b, the bulk orientation is assumed to not have changed during tribological loading. Since the crystal orientation changes observed are much less pronounced in TD (cf. Fig. 2b) than in SD or ND (Fig. 2a, c), the rotational axes of the misorientations accommodated by the DTLs are nearly parallel to TD. The pole figure (PF) provided in Supplementary Fig. 6 corroborates this; the rotational axes of the misorientations left and right of the TB at the lower DTL (arrows 4 and 5 in Fig. 2c) are in close proximity to TD (angular deviation less than 12°). One may therefore conclude that a process of crystal rotation about the TD is at the heart of the sharp transitions in crystal orientation observed at the DTLs in Fig. 2 and labeled as feature (3) in Fig. 3b. This explanation is consistent with previous results not only for copper, but also for steady-state sliding of bronze, and to a certain extent, aluminum or titanium^12,32,33^.

Considering the Hamilton stress tensor components beneath the sliding interface supports this interpretation^21^: All shear stress components with respect to TD (*τ*_SD/TD_ and *τ*_ND/TD_) are approximately zero near the middle of the wear track (i.e. in close proximity to an imaginary axis through the sliding sphere’s center parallel to ND). This is exactly where the TEM foil analyzed in this work was lifted out from. Due to this lack of driving force for dislocation motion perpendicular to TD, a rotation around axes parallel to TD is compatible with Hamilton’s theory. It has to be noted that applying a purely elastic solution for calculation of stress tensors for our experimental conditions is a severe simplification. A misorientation with an axis close to TD concentrated at either DTL has to be accommodated by an arrangement of stationary edge dislocations distributed along the DTL possessing net burgers vectors parallel to ND and perpendicular to TD. An array of exemplary dislocation of this type is presented at the DTL in inset (3a) in Fig. 3b. Although it is likely that still elusive, complex dislocation–dislocation interactions are involved in the DTL formation process, the deposition of stationary dislocations or dislocation structures with such net burgers vectors is necessary as an end result. This is directly supported by the (mis-)orientations measured by ACOM (cf. Fig. 2). In contrast to the two shearing processes, this crystal rotation can thus be observed independently of the TB’s presence.

The suspected dislocation movement necessary for DTL formation may explain the systematically higher misorientation values observed at the segments left of the TB for both upper and lower DTL as opposed to the segments right of the TB. The formation of DTLs has previously been attributed to dislocations with edge components perpendicular to the surface moving down and up from the surface within the sliding sphere’s stress field^4^. Depending on which slip systems experience a high resolved shear stress for a given crystallographic orientation, possible dislocation paths might however also be inclined towards SD. The TB then might act as an obstacle to some of the dislocations possessing a velocity component in SD. If some of these dislocations were entirely blocked at the TB, this would lead to fewer dislocations contributing to the misorientation at the DTLs behind the TB. This is in agreement with what is observed in Fig. 2c. It is important not to confuse such dislocations with the ones accommodating the simple shear process. Their burgers vector direction ideally entails no contribution in ND.

In agreement with this analysis, previous investigations have shown that dislocation-mediated plasticity is a central, elementary mechanism in the formation and evolution of the subsurface microstructures during dry sliding of fcc metals such as copper or nickel^3,19,34^. While the presence of DTLs is therefore expected (cf. Fig. 1), the fact that both DTLs’ characteristics are so similar across the TB (apart from the difference in misorientation) is surprising. Compared to previous results obtained at significantly lower initial Hertzian contact pressures^4,12^, an increased driving force for dislocation motion as well as dislocation–dislocation and dislocation–grain boundary interactions is expected.

At the same time, the effect of crystallographic orientation on friction has been pointed out by numerous investigators and is commonly explained by its influence on plastic deformation^35–37^. The constant depth of the DTLs on both sides of the TB regardless of the different crystal orientations therefore demonstrates the stability of the DTL phenomenon. The model formerly introduced to predict DTL formation may explain this: It is based on the idea of suitable slip systems being available irrespective of crystal orientation, each partially contributing to and in sum accommodating the idealized dislocation motion parallel to ND (and, subordinately, SD) assumed to be responsible for DTL formation^4^. Not the slip system geometry but the stress state is the deciding factor for the depth of the formed DTL(s). This line of reasoning also rationalizes why in the present experiment, ND is not coincident with {111} and SD not coincident with 〈110〉, as might be expected for perfect accommodation of the idealized dislocation movement discussed for the crystal rotation and simple shear processes in this work (cf. Fig. 3b).

This being said, the exact reason for the stability of this phenomenon is still elusive. Nevertheless, some of the microstructural changes observed may be explained by the high contact pressure regime. This includes the formation of two DTLs as opposed to one and the very high misorientation of ~30° accommodated by the lower DTL being complemented by a lower misorientation of only ~7° at the upper DTL. The secondary, upper DTL consequently has to be comprised of fewer dislocations and is speculated to have formed subsequently to the primary, lower DTL to accommodate excess dislocations. These observations as well as the subsurface depths of both DTLs are in agreement with and seamlessly expand on the observations and model for DTL formation in ref. ^4^ (and Supplementary Material therein). Subgrain formation after a single trace may also be attributed to the high load regime and can be interpreted as a precursor for the formation of microcrystalline or nanocrystalline subsurface tribolayers, commonly observed in sliding experiments after an increased number of sliding cycles (e.g. refs. ^12,38,39^).

The observation that instead of spatially distributed and continuous crystal orientation gradients all major misorientations are concentrated at the DTLs (and to a lesser extent at the subgrain boundaries, as seen in Fig. 2a–c) indicates that the formation of such discrete boundaries has to be either energetically or kinematically favorable over sustaining widespread orientation gradients within the plastically strained subsurface area^40^. In the present case, an energetically favorable state may be achieved by both dislocation self-organization into the DTLs and the formation of subgrain boundaries. The areas enclosed within the subgrains are the only ones exhibiting a sizeable orientation gradient in SD and ND (Fig. 2a, c). This is interpreted as a precursor to further sub-division during repeated sliding^12,41^. These observations support the suggestion that a reduction in stored strain energy is a driving force for replacing gradients by discrete misorientation boundaries^3,40,42^.

**Fig. 4:**
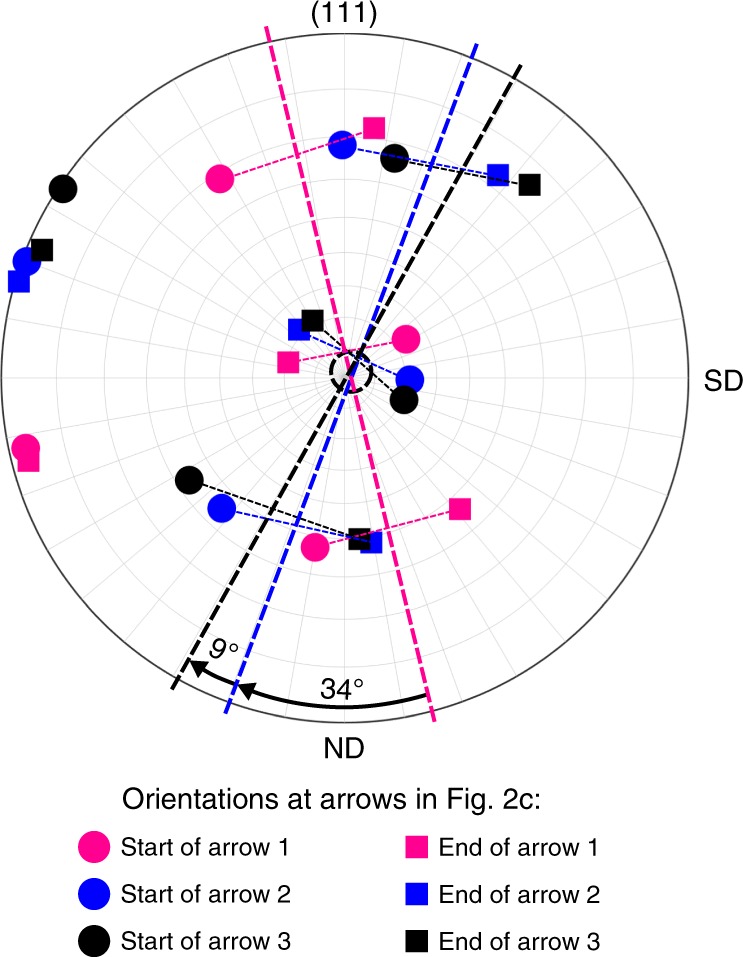
Compatibility analysis of shearing and crystal rotation processes as reflected by the TB. (111) pole figure of orientations left (dot) and right (square) of the TB at arrows 1 (magenta), 2 (blue), and 3 (black) depicted in Fig. 2c. The (manually drawn) dotted lines demark the approximate location of the corresponding twin planes. Their spatial alignment closely matches that of the corresponding TB segments in Fig. 1. The rotations between magenta and blue lines (~34°) as well as blue and black lines (~9°) roughly match the corresponding misorientations at lower and upper DTL as seen in Fig. 2c. The intersection of the three twin plane lines (black circle, manually drawn) shows that the common rotational axis is close to TD (center of the PF). Shearing and crystal rotation processes are both reflected by the TB without contradiction.

Having established the three key processes corresponding to the key features observed in the tribologically altered microstructure (Figs. 1 and 2) and indicated as (1)–(3) in the model schematic in Fig. 3b, their mutual interdependence and compatibility is of special interest. The (111) pole figure presented in Fig. 4 illustrates the relationship between the simple shear and the crystal rotation processes (cf. (1) and (3) in Fig. 3b). The crystal orientations left (dots) and right (squares) of the TB in locations 1–3 (Fig. 2c) are plotted in magenta, blue, and black with respect to the sample coordinate system. The projections of each pair of twin plane orientations marked in the pole figure (dashed lines) exhibit three special characteristics: First, they all show an alignment nearly parallel to the corresponding segments of the TB in Figs. 1 and 2. Second, they are rotated around an axis perpendicular to the projection plane with respect to each other by roughly the same misorientation angles as given in Fig. 2c: 34° in Fig. 4 vs. 33°/28° at arrows 4/5 in Fig. 2c and 9° vs. 8°/5° at arrows 6/7 in Fig. 2c. Third, the intersection of these projection lines (dotted circle in Fig. 4), approximately corresponding to the common rotational axis, is almost coincident with TD. Those characteristics imply that both the changing alignment of the TB plane due to the shearing process, as well as the altered crystal orientations due to the crystal rotation process of the whole areas above both DTLs about an axis parallel to TD agree very well and are without contradiction: The TB demonstrates this compatibility as it reflects both processes.

This insight can be further substantiated assuming that the bulk portion of the TB plane is oriented perpendicular to the drawing plane in Figs. 1 and 2. This assumption is reasonable since the TB appears as a very sharp line in Fig. 1, indicating a small angle between TB plane normal and drawing plane. The Miller indices of the TB plane can be calculated below and above the lower DTL using the crystal orientations left and right of the TB (beginning and end of arrows 1 and 2 in Fig. 2c). The procedure and results are outlined in Supplementary Fig. 7. Below the DTL, the angular deviations between the calculated plane and an ideal {111} twin plane are 3.2° and 4.6° left and right of the TB. Above the TB, those values are 2.1° and 5.6°, respectively. Thus, despite combined shear and crystal rotation, the TB retains its character (misorientation and twin plane) astonishingly well.

Additional evidence of the processes’ compatibility is gained from considering the orientation variations in the vicinity of the TB between upper and lower DTL (Fig. 2). As depicted in Supplementary Fig. 8, there is an orientation gradient with a misorientation of 2.6° and 2.7° over ~200 nm in horizontal direction to the left and to the right of the TB (at the arrows in Supplementary Fig. 8). These strain gradients are suspected to be a result of slip incompatibility at the pre-existing TB during tribologically induced plastic deformation. The observation that both strain gradients possess a small and very similar magnitude of misorientation indicates that despite differently oriented slip systems on both sides of the TB, the need to account for slip incompatibility is small. This supports the proposed processes’ compatibility.

In contrast, the orientation gradients observed in the subgrains (Fig. 2) have to be clearly discriminated from those just discussed. Subgrain formation commonly takes place during sliding as the result of a relaxation process.

This high degree of compatibility observed between the shearing and crystal rotation indicates that the proposed processes depicted in Fig. 3b are physically reasonable. The TB depicted in Supplementary Fig. 4a further suggests generality of the processes discussed above (particularly with regard to normal load).

Finally, considering all three processes, one may speculate that the localization of shear in the subsurface area in conjunction with the observed crystal rotation may be connected to the concept of running-in, frequently observed in tribological systems^43^. As Kuhlmann-Wilsdorf pointed out^44,45^, crystal rotation in the subsurface area may lead to a situation that facilitates easy shear in a steady-state sliding regime, associated with a decreasing coefficient of friction (cf. e.g. ref. ^33^). The presently observed microstructure may be considered as a precursor to a similar situation, entailing two DTLs associated with a lattice rotation around an axis parallel to the TD, as well as substantial simple and localized shear made visible by the TB. It provides invaluable input to the modeling of dislocation motion, combining crystal rotation and shearing underneath a sliding contact. At the same time it demonstrates the stability of DTL formation. As there is general agreement with existing simulations^23^, additional modeling of the associated dislocation transport processes seems very promising and is essential for the quantitative simulation of friction and wear processes.

## Methods

### Sample preparation

Oxygen-free high-conductivity (OFHC) copper plates (Goodfellow, Bad Nauheim, Germany) with a purity higher than 99.95% were used for the unlubricated sliding tests. The plastic deformation caused by the manufacturing process was eliminated by annealing in vacuum at 500 °C for 2 h (average heating rate of 300 K/h) and cooling down to room temperature (average cooling rate 80 K/h). During this process the vacuum was 10^−6^ mbar or better. After grinding with P800, P1200, P2500, and P4000 grit SiC sandpaper (Struers, Willich, Germany), the copper samples were polished with 3 and 1 µm diamond suspension (Cloeren Technology GmbH, Wegberg, Germany). Finally, the copper plates were electropolished with D2 electrolyte (Struers, Willich, Germany). After each preparation step, the specimens were ultrasonically cleaned in isopropanol. This sample preparation process yields an average areal copper surface roughness of *S*_a_ = 80 nm (measured by optical 3D profilometry) and an average grain size of 14 µm (determined using SEM backscatter contrast imaging).

### Experimental setup

The reciprocating tribometer used for the tribological tests was previously described in refs. ^4,46^. As in previous works, sapphire spheres (Saphirwerk, Brügg, Switzerland) with a diameter of 10 mm and an average areal surface roughness *S*_a_ smaller than 5 nm (determined by means of atomic force microscopy) were used as a counter body. The normal load was 26 N yielding an initial Hertzian contact pressure of 1.25 GPa. A single unidirectional sliding pass was performed with a sliding speed of 0.5 mm/s and a stroke length of 12 mm. The humidity and temperature were kept constant at 52% and 22 °C.

### Microstructure and crystallographic orientation analysis

A Helios NanoLab 650 focused ion beam (FIB) system (FEI Company, Hillsboro, OR, USA) was employed to investigate the subsurface microstructure in the vicinity of the TB by lifting a site-specific cross-section from the wear track for scanning transmission electron microscopy (STEM) investigation (high tension 30 kV). By depositing two layers of platinum (SEM followed by FIB), the surface of the copper sample was protected from ion beam damage during FIB micromachining. The TEM foil was cut in the middle of the wear track, perpendicular to the sliding surface and parallel to the sliding direction. TEM foil preparation followed the procedure described by Mayer et al.^47^.

ACOM was performed with precession electron diffraction using the ASTAR system (NanoMegas, Brussels, Belgium) installed on a Tecnai F20 ST (FEI Company, Hillsboro, OR, USA). The TEM was operated at 200 kV in microprobe STEM mode with a nominal probe size of 1 nm and a semiconvergence angle of 1 mrad. Each map was acquired using a probe that scanned the sample with a step size of 1.2 nm and precessed at an angle of 0.5° at each step. The ACOM maps were processed using the ASTAR software package by a template-matching procedure for rapid indexing of spot diffraction patterns adopted from ref. ^48^. After indexing, the resulting maps were corrected for 180° ambiguity^49,50^. Further processing of the crystal orientation data and calculation of misorientations were conducted using custom MATLAB scripts employing the open source toolbox MTEX^51^.

## Supplementary information

Supplementary Information

## Data Availability

The data that support the findings of this study are available under 10.5445/IR/1000100117^13^ and from the corresponding author upon request.

## Code availability

The custom computer code used to evaluate crystal orientation data is available from the corresponding author upon request.
