# Supplementary material for: Early deformation mechanisms in the shear affected region underneath a copper sliding contact
Source: Nat Commun. 2020 Feb 11;11:839. doi: 10.1038/s41467-020-14640-2 (PMC7012857; doi:10.1038/s41467-020-14640-2)
Supplement: Supplementary file 1 — Supplementary Information [file 41467_2020_14640_MOESM1_ESM.pdf]

Supplementary Information

Early deformation mechanisms in the shear affected region underneath a copper sliding contact

Haug et al.

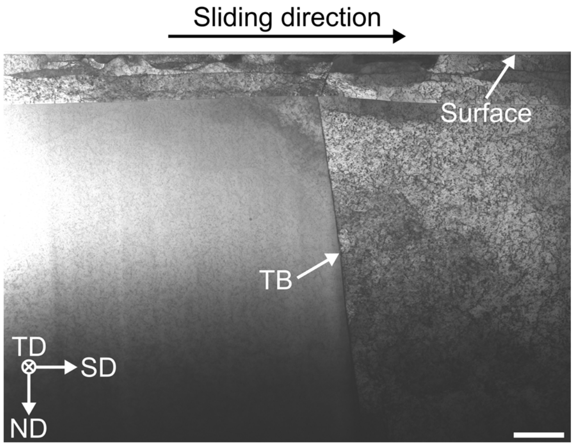

Supplementary Figure 1: Tribologically deformed microstructure. STEM BF image acquired at 30 kV high tension of the area surrounding the TB. The scale bar corresponds to 500 nm.

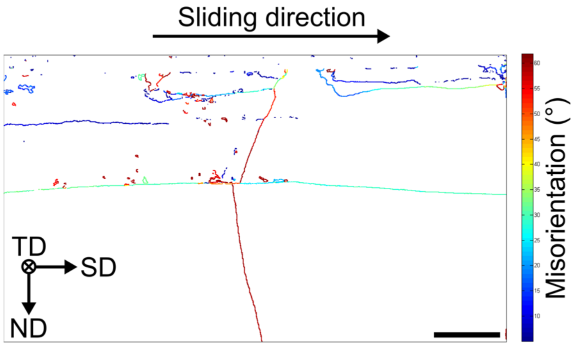

Supplementary Figure 2: Color coded boundary misorientation for grains calculated with a segmentation angle of 5° from ACOM orientation data. The scale bar corresponds to 250 nm.

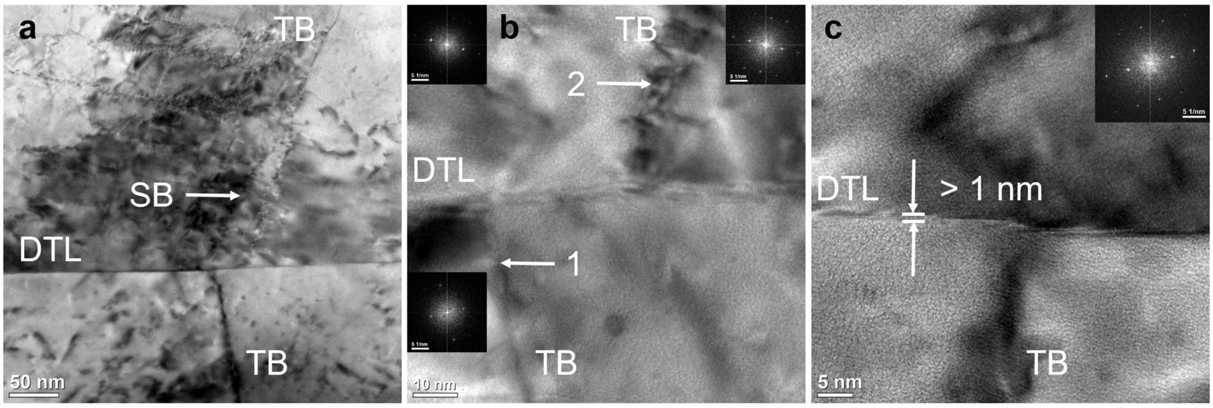

Supplementary Figure 3: High-resolution TEM (HRTEM) measurements directly beneath the surface, containing the intersections between the DTLs and the twin boundary (TB). HRTEM imaging was performed using an aberration corrected Titan 80-300 (FEI Company, Hillsborough, Oregon, USA) operated at 300kV and equipped with a US1000 slow scan CCD camera (Gatan, Pleasanton, CA, USA). **a** As indicated by an arrow (SB), additional subgrain boundaries are visible above the lower DTL and parallel to TB. **b** The TB above the lower DTL (arrow 2) is much more poorly defined than below (arrow 1). **c** The lower DTL is very well defined from the TEM images; a bit more than 1 nm wide.

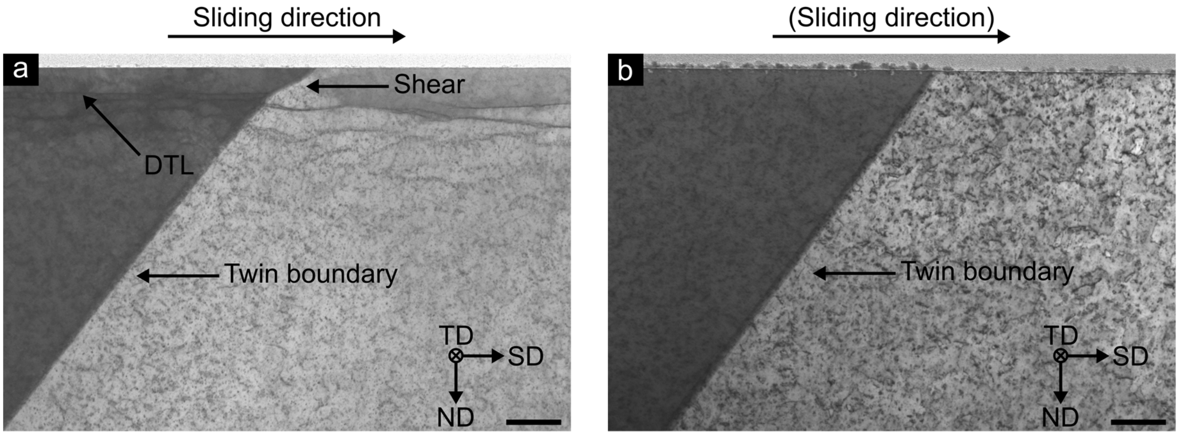

Supplementary Figure 4: Comparison of the microstructure in the vicinity of a copper twin boundary after tribological loading in the center of the wear track and in an unaffected region next to the wear track. The experimental parameters used are consistent with those presented in the methods section of the main text with the exception of a reduced normal load (1 N) and different material (oxygen-free electronic grade (OFE) copper, annealed in air for 10 minutes at 800°C to achieve an average grain size of approximately 80 µm), containing a number of annealing twins. **a** STEM image of a TEM foil located in the center of the wear track parallel to the direction of sliding, i.e. similarly to the TEM foil presented in the manuscript. A DTL in both grains as well as a shear of the twin boundary above the DTL is visible. **b** STEM image of a TEM foil cut parallel to the foil in **a** at the same twin boundary, but outside of the wear track. Both scale bars correspond to 200 nm.

It is clearly visible that the twin boundary extends all the way to the sample surface outside of the tribologically deformed area seen in a. In conjunction, images a and b are being considered added proof that the assumption in the main text regarding the twin boundary's preexistence and straight line shape are reasonable.

Furthermore, a suggests generality of the processes discussed in the main text: For a much lower normal load of 1N, a slightly altered material (OFE copper) and a different inclination angle of the TB towards ND ($\alpha \approx 39°$, cf. main text), the simple shear process as well as DTL formation can still be discerned. According to expectation (cf. Ref. 4 in the main text) the DTL is observed at a lower average depth of only roughly 115 nm and the simple shear is less pronounced with a tilt angle of only approx. 22° with respect to the bulk, presumably owing to the reduced driving forces for dislocation movement.

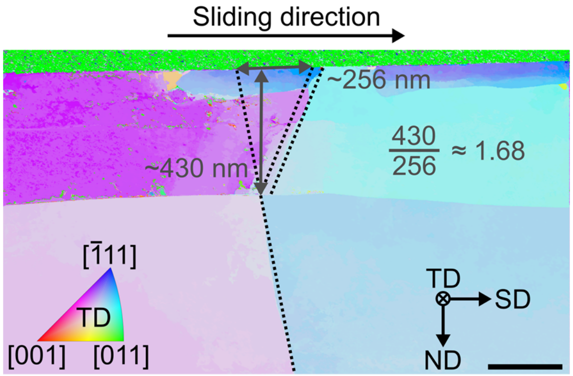

Supplementary Figure 5: Quantification of simple shear process. Dividing the vertical distance between interface and the lower DTL (~430 nm) by the distance that the TB was displaced in SD from its estimated original position at the sliding interface (~256 nm) yields a value of 430 nm / 256 nm ≈ 1.68. This is in agreement with evaluating the angle between the TB segment in the bulk and in the sheared area (1 / tan(30°) ≈ 1.73, see main text). The scale bar corresponds to 250 nm.

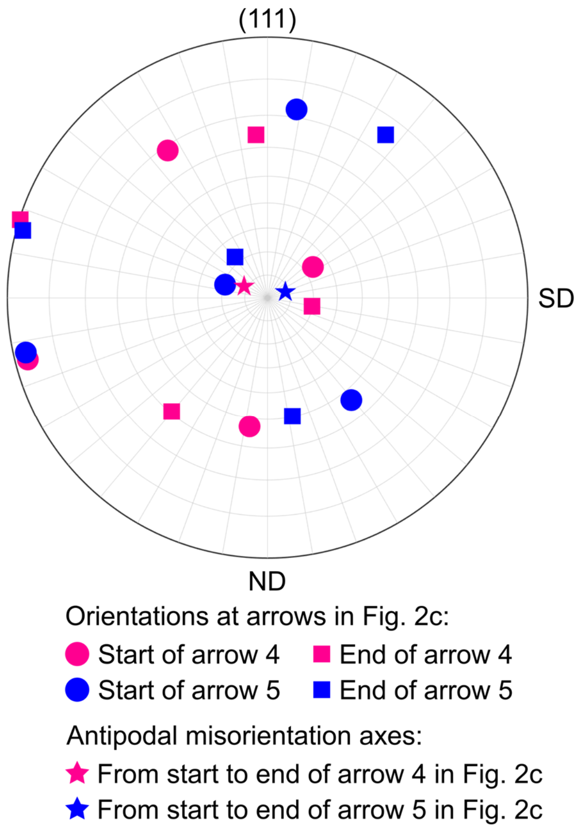

Supplementary Figure 6: Analysis of crystal rotation process at lower DTL. (111) pole figure (PF) of orientations below (dot) and above (square) the lower dislocation trace line at positions left (magenta) and right (blue) of the TB (arrows 4 and 5 in Fig. 2c). The corresponding antipodal misorientation axes (magenta and blue star, calculated in specimen coordinates) are in close proximity to TD (at the center of the PF).

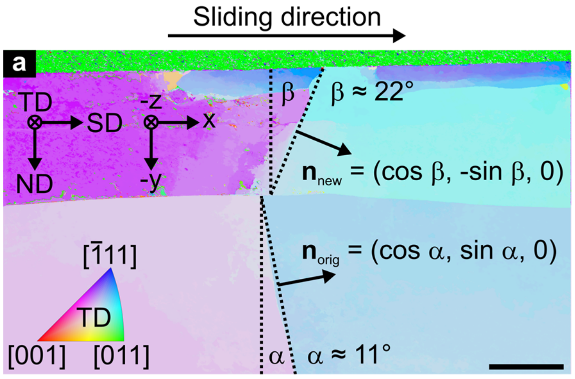

Supplementary Figure 7: Calculation of tilted TB segment interface plane Miller indices under the assumption that TB plane is orthogonal to drawing plane. a Angles and equations used for calculation of plane normal vectors in specimen coordinate system xyz below ($\mathbf{n}_{orig}$) and above ($\mathbf{n}_{new}$) lower DTL. **b** Rounded Miller indices calculated for interface plane normal vectors below lower DTL ($\mathbf{n}_{orig}$, points 1A and 1B) and above lower DTL ($\mathbf{n}_{new}$, points 2A and 2B) left and right of the TB using the crystallographic orientation at the corresponding points indicated by arrows. Angles specified correspond to smallest angular misorientation angle between calculated Miller indices and <111> at each location, considering crystallographic symmetry. Both scale bars correspond to 250 nm.

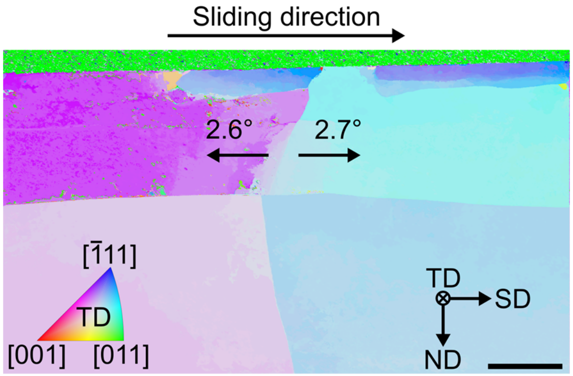

Supplementary Figure 8: Quantification of orientation gradients next to the TB between upper and lower DTL. The values of 2.6° and 2.7° constitute the misorientation between the crystallographic orientations measured by ACOM at the start and end of each arrow. The scale bar corresponds to 250 nm.
